# High-Wattage Pulsed Irradiation of Linearly Polarized Near-Infrared Light to Stellate Ganglion Area for Burning Mouth Syndrome

**DOI:** 10.1155/2014/171657

**Published:** 2014-10-19

**Authors:** Yukihiro Momota, Koichi Kani, Hideyuki Takano, Fumihiro Matsumoto, Keiko Aota, Daisuke Takegawa, Tomoko Yamanoi, Chika Kondo, Shigemasa Tomioka, Masayuki Azuma

**Affiliations:** ^1^Department of Oral Medicine, Institute of Health Biosciences, the University of Tokushima Graduate Faculty of Dentistry, Kuramoto 3-18-15, Tokushima 770-8504, Japan; ^2^Department of Dental Anesthesiology, Institute of Health Biosciences, the University of Tokushima Graduate Faculty of Dentistry, Kuramoto 3-18-15, Tokushima 770-8504, Japan

## Abstract

The purpose of this study was to apply high-wattage pulsed irradiation of linearly polarized near-infrared light to the stellate ganglion area for burning mouth syndrome (BMS) and to assess the efficacy of the stellate ganglion area irradiation (SGR) on BMS using differential time-/frequency-domain parameters (D parameters). Three patients with BMS received high-wattage pulsed SGR; the response to SGR was evaluated by visual analogue scale (VAS) representing the intensity of glossalgia and D parameters used in heart rate variability analysis. High-wattage pulsed SGR significantly decreased the mean value of VAS in all cases without any adverse event such as thermal injury. D parameters mostly correlated with clinical condition of BMS. High-wattage pulsed SGR was safe and effective for the treatment of BMS; D parameters are useful for assessing efficacy of SGR on BMS.

## 1. Introduction

Burning mouth syndrome (BMS) is mainly characterized by oral burning or painful sensations without significant pathological changes in the oral mucosa [1]. Relationships between BMS and the function of the autonomic nervous system (ANS) have also been pointed out recently [2–4]. Stellate ganglion near-infrared irradiation (SGR), which is considered to correct abnormalities in the ANS, has been reported to be beneficial in BMS patients [2, 3], but the potency of SGR on ANS is thought to be lower than that of stellate ganglion block (SGB), the treatment of choice for most pain clinicians. One of the strategies to enhance the curative effects is to raise the wattage levels of SGR. High-wattage SGR uses high-energy infrared light and has a high level of irradiation depth in the irradiated tissue. However, it is well known that high-energy infrared light easily causes thermal injury at the irradiation site due to the heat that is evolved when it is absorbed in body water [5]. Thus, a pulsed irradiation method for high-wattage SGR was developed in 2009 to enhance curative effects and reduce adverse events such as thermal injury. The biggest advantage of this method is to prevent heat accumulation at the site by pulsed irradiation.

Heart rate variability (HRV) analysis, which involves measurement of beat-to-beat variations in heart rate, is useful to assess autonomic activity and to diagnose autonomic neuropathy without subjecting patients to any stress [6, 7]. We found that previous parameters of HRV analysis did not reflect the clinical condition of BMS patients and developed new parameters, that is, differential time-/frequency-domain parameters (D parameters) [3]. The D parameters were defined as the differential between the previous parameters of just before and after irradiation and were considered to index the autonomic response [3].

In this study, we applied high-wattage pulsed SGR on BMS and assessed the efficacy of high-wattage pulsed SGR on BMS using D parameters.

## 2. Case Reports

**Figure 1:**
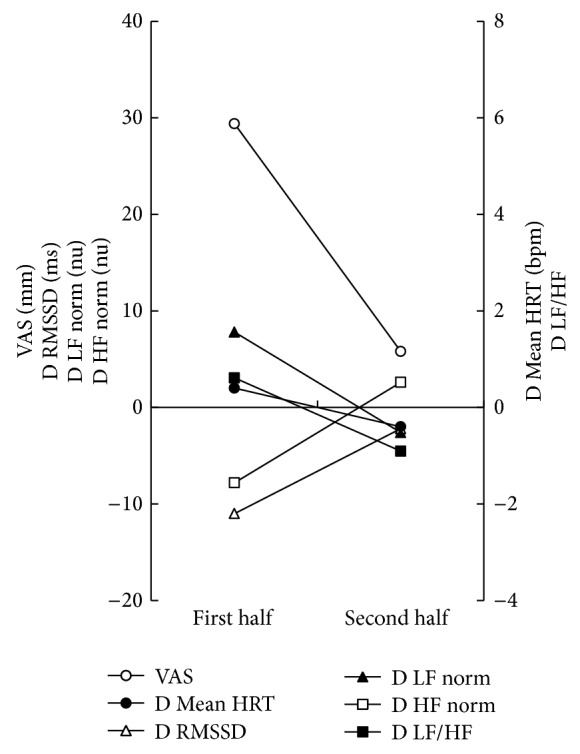
Time course of differential frequency-/time-domain parameters during SGR in case 1.

*Case 1*. The patient was a 76-year-old Japanese female who attended the Department of Oral Medicine, Tokushima University Hospital because of spontaneous glossalgia, intraoral sticky feeling, dry mouth feeling, and amblygeustia. She was on medication (valsartan: 40 mg/day) for hypertension. She was given a diagnosis of BMS according to the following criteria: (1) presence of pain or a burning sensation on the surface of the tongue; (2) absence of local or systemic disease related to the above tongue symptoms, such as candidiasis, xerostomia, glossitis, anemia, neuralgia, diabetes mellitus, and referred pain from dentalgia; (3) absence of somatization of a psychiatric disorder; and (4) absence of pain medication. She received high-wattage pulsed SGR (SUPER LIZER PX Type 2; Tokyo Iken Co., Ltd., Tokyo, Japan) under the following conditions: power of 5.0 W, pulse width of 3 ms, interpulse period of 7 ms, duration of 3 min, and once a week for 10 weeks. The response to SGR was evaluated by a visual analogue scale (VAS: 0–100 mm) representing glossalgia intensity and HRV analysis (SA-3000P; Tokyo Iken Co., Ltd.) just before and after every irradiation. The following D parameters were adopted for HRV analysis: differential mean heart rate (D Mean HRT), differential root mean square of successive NN interval differences (D RMSSD), differential normalized low frequency (D LF norm), differential normalized high frequency (D HF norm), and differential low-frequency/high-frequency ratio (D LF/HF). The mean value of the parameters was calculated by averaging 5 values each during the first and second half of the total treatment period, with each half consisting of 5 treatments in 5 weeks. The mean values of VAS, D Mean HRT, D LF norm, and D LF/HF ratio decreased from 29.4 mm to 5.8 mm, 0.4 bpm to −0.4 bpm, 7.8 nu to −2.6 nu, and 0.612 to −0.906, respectively (Figure 1). The mean values of D RMSSD and D HF norm increased from −11.0 ms to −2.2 ms and −7.8 nu to 2.6 nu, respectively (Figure 1). The patient completed the course of SGR without feeling any discomfort such as heat pain.

**Figure 2:**
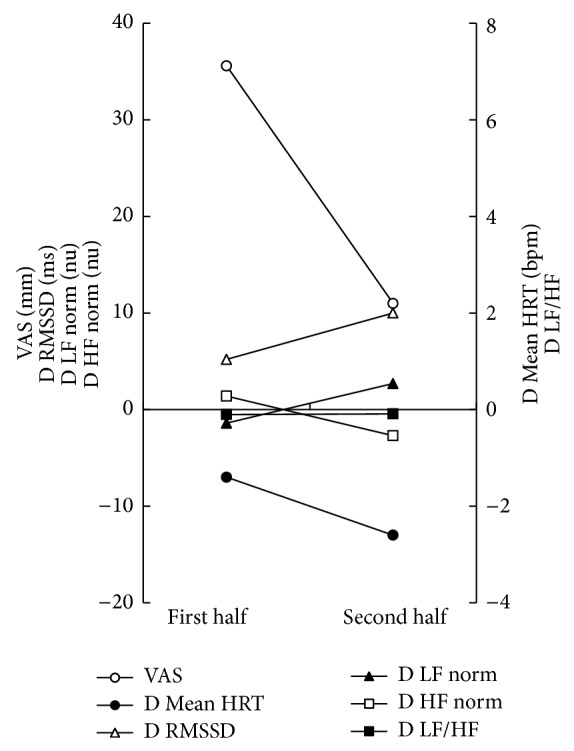
Time course of differential frequency-/time-domain parameters during SGR in case 2.

*Case 2*. The patient was a 72-year-old Japanese female attending our hospital because of spontaneous and induced glossalgia. She was in treatment for hyperlipidemia, insomnia, and chronic gastritis. She was given a diagnosis of BMS according to the same criteria as case 1 and received high-wattage pulsed SGR under the same conditions as case 1. The mean values of VAS, D Mean HRT, and D HF norm decreased from 35.6 mm to 11.0 mm, −1.4 bpm to −2.6 bpm, and 1.4 nu to −2.7 nu, respectively (Figure 2). The mean values of D RMSSD, D LF norm, and D LF/HF ratio increased from 5.2 ms to 10.0 ms, −1.4 nu to 2.7 nu, and −0.102 to −0.088, respectively (Figure 2). She completed the course of SGR without feeling any discomfort such as heat pain.

**Figure 3:**
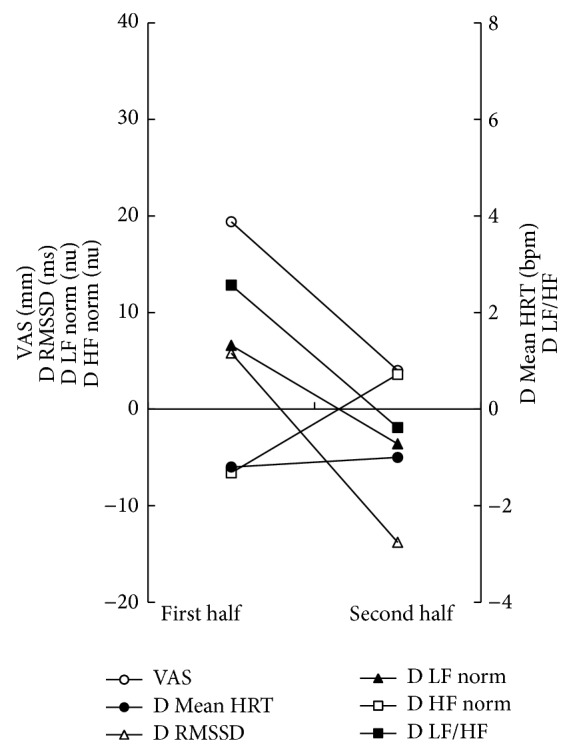
Time course of differential frequency-/time-domain parameters during SGR in case 3.

*Case 3*. The patient was a 65-year-old Japanese female attending our hospital because of spontaneous and induced glossalgia. She was in treatment for insomnia and cystitis. She was given a diagnosis of BMS and received high-wattage pulsed SGR like cases 1 and 2. The mean values of VAS, D RMSSD, D LF norm, and D LF/HF ratio decreased from 19.4 mm to 4.0 mm, 5.8 ms to −13.8 ms, 6.6 nu to −3.6, and 2.567 to −0.383, respectively (Figure 3). The mean values of D Mean HRT and D HF norm increased from −1.2 bpm to −1.0 bpm and −6.6 nu to 3.6 nu, respectively (Figure 3). She completed the course of SGR without feeling any discomfort such as heat pain.

The study was approved by the Institutional Review Board and Medical Ethics Committee of Tokushima University Hospital. All patients provided their informed consent after a full explanation of all procedures.

## 3. Discussion

To our knowledge, this is the first report of cases in which high-wattage pulsed SGR was applied to BMS, and the efficacy of high-wattage pulsed SGR on BMS was assessed using D parameters. High-wattage pulsed SGR relieved glossalgia of BMS patients without any adverse events such as thermal injury. Throughout the continuum of this study, we observed three types of BMS in terms of its response to SGR. In fact, both differential time- and differential frequency-domain parameters reflected clinical conditions (Table 1). With regard to differential frequency-domain parameters, D LF norm, D HF norm, and D LF/HF ratio correlate with each other because each original parameter, LF norm, HF norm, and LF/HF ratio, is mathematically defined in a mutually complementary way as follows: LF norm or HF norm = 100 × LF or HF/(total power − very low frequency) [7, 8]. For example, an increase in the D HF norm means increase in R-R interval of high-frequency wave and indexes parasympathetic activation [3], whereas the extension of R-R intervals helps shift a high-frequency trend to a low-frequency trend and can be considered as opposing parasympathetic activation. With regard to differential time-domain parameters, RMSSD, from which D RMSSD is obtained, means successive differences at adjacent R-R intervals and essentially represents ever-changing autonomic conditions [9]. Therefore, D RMSSD easily reflects autonomic responsiveness because it is defined as differential before and after some intervention such as SGR. However, RMSSD does not show a negative value but a positive value by mathematical definition; whether a change to sympathetic tone or parasympathetic tone occurs cannot be distinguished by the results of D RMSSD alone. Mean HRT, which is obtained from heart rate, is extensively utilized to index how the ANS, particularly the parasympathetic nerve, responds to various stimuli [10, 11]. Hence, collective evaluation with both D RMSSD and D Mean HRT is necessary for this purpose. Accordingly, time-domain measurements using D RMSSD and D Mean HRT may be more advantageous for analysis of autonomic response than frequency-domain measurements using D LF norm, D HF norm, and D LF/HF ratio. However, we do not currently have enough data to determine which is better for the purpose, time- or frequency-domain measurements. Continuous collection of data and further investigation are required to clarify that question.

## 4. Conclusion

We confirmed that high-wattage pulsed SGR was safe and effective for the treatment of BMS. We verified that D parameters correlated with clinical condition of BMS and suggested that D parameters were useful for assessing the efficacy of SGR for BMS.

## Figures and Tables

**Table 1:** Fluctuation of differential frequency-/time-domain parameters during SGR.

	Case 1	Case 2	Case 3
VAS	↓	↓	↓
D Mean HRT	↓	↓	↑
D RMSSD	↑	↑	↓
D LF norm	↓	↑	↓
D HF norm	↑	↓	↑
D LF/HF	↓	↑	↓

VAS: visual analogue scale; D Mean HRT: differential mean heart rate; D RMSSD: differential root mean square of successive NN interval differences; D LF norm: differential normalized low frequency; D HF norm: differential normalized high frequency; D LF/HF: differential low-frequency/high-frequency ratio.

↑: increase; ↓: decrease.
